# Comparison of Primary and Secondary Prophylaxis Using PEGylated Recombinant Human Granulocyte–Stimulating Factor as a Cost-Effective Measure in Malignant Neoplasms: A Multicenter Retrospective Study

**DOI:** 10.3389/fphar.2021.690874

**Published:** 2021-10-29

**Authors:** Qiuji Wu, Qiu Li, Jun Zhang, Zhumei Luo, Jin Zhou, Jing Chen, Yong Luo

**Affiliations:** ^1^ Department of Medical Oncology, Cancer Center, West China Hospital, Sichuan University, Chengdu, China; ^2^ Department of Oncology, The Third People’s Hospital of ChengduChengdu, China; ^3^ Department of Medical Oncology, Sichuan Cancer HospitalChengdu, China; ^4^ Department of Head and Neck Oncology, West China Hospital, Sichuan University, Chengdu, China

**Keywords:** real-world, cost-effectiveness, PEGylated recombinant human granulocyte-stimulating factor, febrile neutropenia, prophylaxis

## Abstract

**Purpose:** The aim of the study was to evaluate the cost-effectiveness of PEGylated recombinant human granulocyte–stimulating factor (PEG-rhG-CSF) as a means of achieving primary and secondary prophylaxis against chemotherapy-induced neutropenia cancer cases.

**Methods:** Individuals who underwent PEG-rhG-CSF therapeutics were monitored for 12 months, together with thorough examination of individual medical records for extracting medical care costs. Both prophylaxis-based therapeutic options (primary/secondary) were scrutinized for cost-effectiveness, using a decision-making analysis model which derived the perspective of Chinese payers. One-way and probabilistic sensitivity analyses were used to assess the robustness of the model.

**Results:** In summary, 130 clinical cases treated using PEG-rhG-CSF prophylaxis were included in this study: 51 within the primary prophylaxis (PP) group and 79 within the secondary prophylaxis (SP) group. Compared with SP, PP-based PEG-rhG-CSF successfully contributed to a 14.3% reduction in febrile neutropenia. In general, PP was estimated to reduce costs by $4,701.81 in comparison to SP, with a gain of 0.02 quality-adjusted life years (QALYs). Equivalent results were found in differing febrile neutropenia (FN) risk subgroups. Sensitivity analyses found the model outputs to be most affected for the average time of hospitalization and for the cost of FN.

**Conclusion:** From the perspective of Chinese payers, PP with PEG-rhG-CSF should be considered cost-effective compared to SP strategies in patients who received chemotherapy regimens with a middle- to high-risk of FN.

## Introduction

Chemotherapy-induced febrile neutropenia (FN) is a main repercussion derived from myelosuppression stemming from chemotherapeutic measures (Aarts et al., 2013). FN can lead to lowering dose limits of chemotherapy drugs, delayed chemotherapy, and severe infections. Such repercussions typically increase treatment costs, together with reducing the efficacy of chemotherapy and quality of survival, affecting patient prognosis and possibly even leading to death (Gisselbrecht et al., 1997; Tirelli et al., 1998). Therefore, primary/secondary prophylaxis measures employing granulocyte colony–stimulating factor (G-CSF) were recommended by national and international guidelines as a measure for reducing FN development within the most vulnerable patients incurring elevated risks of FN (Aapro et al., 2010; Langford and Chrisp, 2010; Smith et al., 2015). FN risk assessments are based upon multiple patient parameters, including demographic data, and primary disease parameters (such as tumor progression status), together with therapeutic parameters such as the degree of aggressiveness in chemotherapy approaches on the patient. Consequently, prophylaxis protocols that rely solely on the type and intensity of chemotherapy regimens can result in exposing patients to a greater number of FN events.

Currently, the effective and widely used drug for treating chemotherapy-induced febrile granulocytopenia is recombinant human granulocyte colony–stimulating factor (rhG-CSF) (Kuderer et al., 2007; Wang et al., 2015). However, rhG-CSF usually requires multiple doses to achieve improvement, leading to exacerbated drug-related adverse effects, which undoubtedly prolongs the treatment cycle and increases the physical and psychological stress in patients (Kuwabara et al., 1996; Bhana, 2007). PEGylated recombinant human granulocyte–stimulating factor (PEG-rhG-CSF) is a chemically modified version of rhG-CSF using monomethoxy polyethylene glycol, which has the characteristic of relieving neutrophil deficiency with a single dose (Holmes et al., 2002; Green et al., 2003; Kubo et al., 2016; Cerchione et al., 2017). Based on its convenience, stability and efficacy, long-acting PEGylated rhG-CSF is recommended by relevant treatment guidelines and widely used in clinical practice (Aapro et al., 2010; The society of chemothera, 2019; Network.linical Pra, 2020).

Increasingly, cost-effectiveness analysis is used to compare the costs and health outcomes of different interventions to provide a basis for policy decisions. Moreover, long-acting PEGylated rhG-CSF has been highlighted in previous pharmacoeconomic studies as being cost-effective (Lyman et al., 2009; Sebban et al., 2012; Perrier et al., 2013). Consequently, further research on the cost-effective benefits of population-based primary or secondary prophylaxis with PEG-rhG-CSF is essential to optimize medical resource allocations for chemotherapy-induced FN. Although several investigations have explored the cost/medical benefit of PEGylated rhG-CSF for primary versus secondary prophylaxis for oncology patients in multiple countries (Ramsey et al., 2009; Fust et al., 2014; Hill et al., 2014; Fust et al., 2017), most of these studies were based on published clinical trials and were limited to specific oncology patients, leading to a paucity of data in the real world. Furthermore, to our knowledge, no relevant studies have explicitly evaluated the cost-effectiveness of PEG-rhG-CSF primary versus secondary prophylaxis in Chinese cancer patients. Due to regional and health insurance plan limitations, available cost-effectiveness data from Western countries may not be generalizable to Asian populations.

The investigation described later analyzed the cost-effectiveness of PEG-rhG-CSF as primary and secondary prophylactic measures against FN from the perspective of Chinese payers. This was achieved by the use of the hospital information system (HIS) database of several hospitals in southwestern China from 2015 to 2019, with the intention of evaluating the economics of PEG-rhG-CSF and providing real-world evidence for rational and economical clinical deployment of this drug.

## Materials and Methods

### Data Collection

This study was accepted by the Biomedical Ethics Committee of West China Hospital of Sichuan University, without the requirement for informed, written consent by patients. In this analysis, medical records from three hospitals in southwest China, from 2015 to 2019, were retrospectively collected to assess the effectiveness, together with costs, for PEG-rhG-CSF therapies in oncology patients. The criteria for including patients into this study were as follows: a) adult age (18–75 years); b) malignancy diagnosed by pathological histology or cytology; c) receiving chemotherapy with medium- to high-risk FN regimen; and d) having received PEG-rhG-CSF as primary or secondary prophylaxis during chemotherapy. Exclusion criteria were as follows: a) bone marrow metastasis and b) no data on absolute neutrophil count after granulocyte-stimulating factor prophylaxis or treatment.

### Model Construction

Both prophylaxis-based therapeutic options (primary/secondary prophylaxis) were scrutinized for cost-effectiveness, using a decision-making analysis model derived from Chinese payer’s perspective. For the purpose of this study, primary prophylaxis (PP) was defined as the PEG-rhG-CSF therapy commencing at the start (1^st^) of the chemotherapeutic cycle (or in a previous cycle without a neutropenic event). Secondary prophylaxis (SP) was defined for PEG-rhG-CSF therapy that was commenced following a neutropenic event immediately preceding the chemotherapeutic cycle. Clinical effectiveness was measured by neutropenic events, including neutropenia without fever or infection and FN (defined as neutropenia combined with fever and/or infection). A log of neutropenic incidents was recorded for every patient during each chemotherapy cycle. The model creation and analysis were carried out using TreeAge software (TreeAge Pro^®^ statistical package 2020 R1, Williamstown, Massachusetts, United States), SPSS software 26 (IBM, Armonk, New York, United States), and Microsoft Excel 2019 (Microsoft, Redmond, Washington, United States). Each model chemotherapy cycle constituted 4 weeks (1 month) across a 12-month time horizon, matching this study’s follow-up time. The primary outcome for this investigation was the incremental cost-effectiveness ratio (ICER; a ratio-based the difference of cost and effectiveness variations between PP and SP therapeutics). Consequently, the ICER value was evaluated against the willingness to pay (WTP; highest price a patient is comfortable to pay for an individual QALY). Based on the WHO research and guidelines, the WTP for this specific study was set at three-fold the individual Chinese citizen’s GDP (USD 30,313.52-2019) (Molassiotis et al., 2002; Eichler et al., 2004; Murray et al., 2000; O (2002). The world hea, 2002).

### Cost and Utilities

The cost and cost-effectiveness were conducted from the Chinese payer system perspective but were limited to direct medical cost only. Such costs include G-CSF, PEG-rhG-CSF, and antibiotic therapies, laboratory tests and hospitalization costs. Direct medical cost evaluation was collected from centralized procurement data in the Chinese market, Hospital Information System, and published the literature (Table 1). Since multiple tumor types and various treatment options were included in the study, the cost for chemotherapy drugs was not taken into consideration. All costs were recorded in USD (February 15, 2021 exchange rate, 6.9851 RMB = 1 US dollar) (Sigsgaard et al., 2001).

**TABLE 1:** Model parameters and assumptions.

Variable	Value (range)	Distribution	Source
**Cost (USD)**			
PEG-rhG-CSF	244.09 (170.86–317.32)	Fix	Pater et al. (1994)
rhG-CSF	27.70 (19.39–36.01)	Fix	Rojas et al. (2014)
Hospitalization	70.88 (49.62–92.15)	Γ	Local estimate
Test	196.70 (137.69–255.72)	Γ	Local estimate
FN hospitalization cost	7,158.10 (5,010.67–9,305.52)	Γ	Xia et al. (2020)
Post-hospitalization FN cost	4,772.07 (3,340.45–6,203.69)	Γ	Weycker et al. (2008)
**Utility values**			
On chemotherapy	0.70	Β	Ramsey et al. (2009)
FN	0.33	Β	Ramsey et al. (2009)
SN	0.42	Β	Gold et al. (2009)
Grade 1–2 Neutropenia	0.69	Β	Nafees et al. (2008)
**Others**			
Discount rate	0.03 (0–0.05)	-	Salgia et al. (2018)
Hospitalization days in PP	6.10 ± 3.66	-	Local estimate
Hospitalization days in SP	5.28 ± 3.73	-	Local estimate
Hospitalization days for FN in PP	9.48 ± 3.06	-	Local estimate
Hospitalization days for FN in SP	9.52 ± 2.67	-	Local estimate

PEG-rhG-CSF, PEGylated recombinant human granulocyte-stimulating factor; rhG-CSF, recombinant human granulocyte colony–stimulating factor injection; FN, febrile neutropenia; SN, severe neutropenia; PP, primary prophylaxis; and SP, secondary prophylaxis.

### Sensitivity Analysis

A series of deterministic sensitivity analyses were conducted to explore whether primary PEG-rhG-CSF prophylaxis is cost-effective when the cost, utility values, and other parameters of PEG-rhG-CSF vary over a broader range. The variables in the deterministic sensitivity analysis varied within confidence intervals or ±30%. Based on the distribution characteristics of each parameter, a γ distribution was used for the cost parameter, and a β distribution was used for the health utility values. Then, we performed 1,000 Monte Carlo simulations for probabilistic sensitivity analysis. The univariate sensitivity analysis results were expressed as tornado plots, and the probabilistic sensitivity analysis results were expressed as cost–benefit acceptability curves. We also analyzed the possibility of primary PEG-rhG-CSF prophylaxis being cost-effective in the subgroups with different FN risks.

## Results

### Patient Datasets

One hundred thirty (130) patients fulfilling the inclusion and exclusion criteria for our study, of which 51 participants underwent PEG-rhG-CSF PP, while 79 participants underwent PEG-rhG-CSF SP. In the PP group, a total of 68.6% of patients received chemotherapeutic cycles with an FN risk being >20%, while in the SP group, this percentage was 45.6%. Among PP patients, 21.6% had metastatic cancer, compared with 21.5% of patients with metastatic cancer in the SP group. Baseline characteristics of the PEG-rhG-CSF–treated patients are presented in Table 2.

**TABLE 2:** Patient characteristics and results of the study.

Characteristic	Primary prophylaxis (*N* = 51)	Secondary prophylaxis (N = 79)	*p* value
**Age (mean ± SD)**	51.5 ± 14.1	50.7 ± 11.6	0.630
>65 years, *n* (%)	15 (29.4)	12 (15.2)	0.051
**Gender, *n* (%)**			0.115
Male	21 (41.2)	22 (27.8)	
Female	30 (58.8)	57 (72.2)	
**Cancer type, *n* (%)**			<0.001
Breast cancer	15 (29.4)	41 (51.9)	
Lymphoma	24 (47.1)	20 (25.3)	
Nasopharyngeal carcinoma	6 (11.8)	3 (3.8)	
Non–small-cell lung cancer	0	2 (2.5)	
Gastrointestinal cancers	0	8 (10.2)	
Pancreatic cancers	0	3 (3.8)	
Sarcoma	3 (5.9)	0	
Others	3 (5.9)	2 (2.5)	
**Primary overall stage, *n* (%)**			0.051
Nonmetastatic phase	35 (68.6)	61 (77.2)	
Metastatic phase	11 (21.6)	17 (21.5)	
Unknown	5 (9.8)	1 (1.3)	
**Risk for febrile neutropenia**			0.010
>20%	35 (68.6)	36 (45.6)	
10–20%	16 (31.4)	43 (54.4)	
**aCCI (mean ± SD)**	2.97 ± 2.222	2.33 ± 2.02	0.163
0	6	16	
1–2	19	36	
3–5	19	20	
>5	7	7	
**Others factors, *n* (%)**			0.582
Previous therapies	2 (3.9)	4 (5.1)	
Surgery	32 (62.7)	61 (77.2)	
Diabetes	2 (3.9)	5 (6.3)	
Hypertension	8 (15.7)	7 (8.9)	
Smoking	10 (19.6)	12 (15.2)	
**No. of patients with FN, *n* (%)**			<0.001
Cycle 1	3 (5.9)	42 (53.2)	
Cycle 2	4 (7.8)	20 (25.3)	
Cycle 3	0	12 (15.2)	
Cycle 4	2 (3.9)	4 (5.1)	
Cycle 5	0	6 (7.6)	
Cycle 6	2 (3.9)	2 (2.5)	
Overall incidence	6 (11.8)	62 (78.5)	

aCCI, age-adjusted Charlson Comorbidity Index; FN, febrile neutropenia; SD, standard deviation.

### Economic Evaluation

**FIGURE 1:**
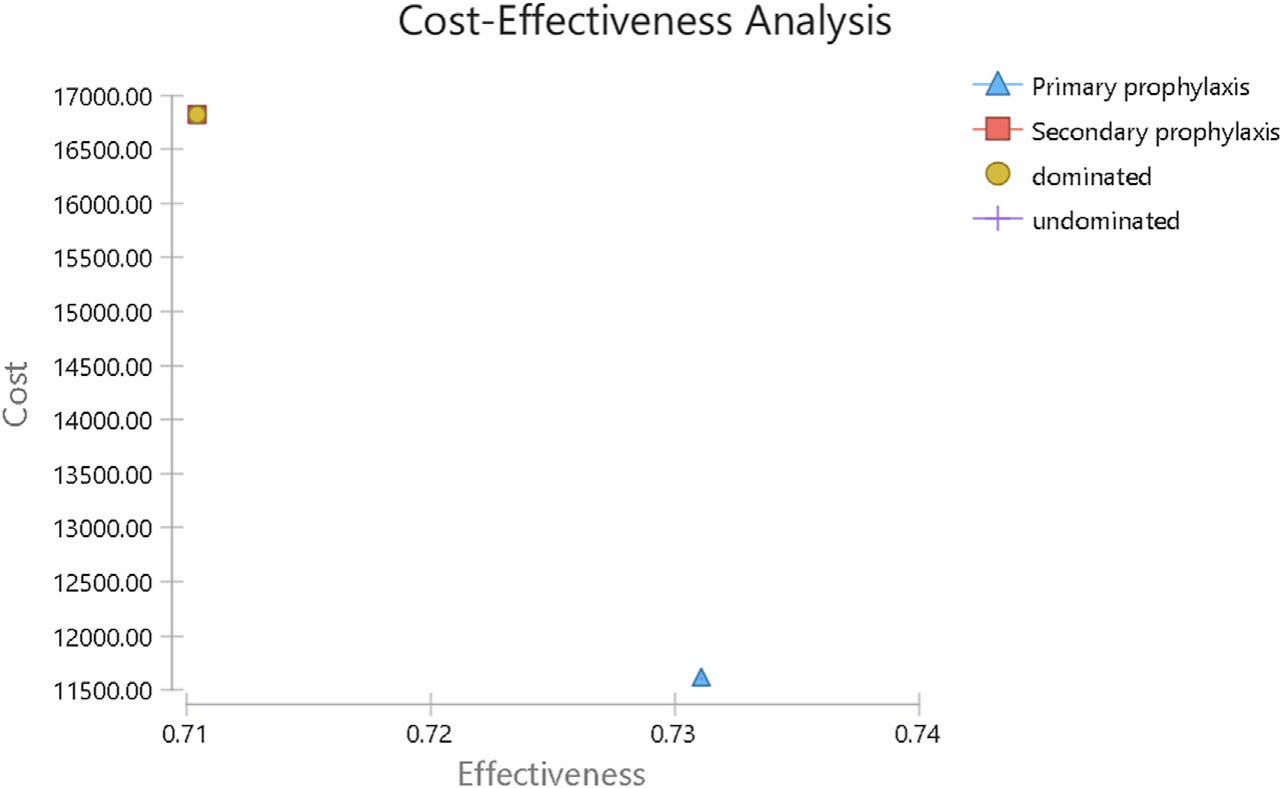
Incremental cost-effectiveness ratio of cost-effectiveness analysis.

In the PP arm, 6 of 51 patients (11.8%) suffered a minimum of one FN event, while in the SP arm, FN afflicted 62 out of 79 patients (78.5%). From Chinese payers’ perspective, the average cost of primary PEG-rhG-CSF prophylaxis is $11,611.62, with an average gain of 0.73 QALYs per patient over a 1-year time horizon. The average cost of secondary PEG-rhG-CSF prophylaxis was $16,313.43, with an average gain of 0.71 QALYs (Figure 1). For all patients in this study, PEG-rhG-CSF as primary prophylaxis dominated all comparators on FN events avoided and QALYs gained. Consensus results were also observed in the subgroup analysis of patients who received chemotherapy regimens with a risk of FN greater than 20% and patients who received chemotherapy regimens with an FN risk of 10–20%(Table 3).

**TABLE 3:** Base case and subgroups cost-effectiveness results for PEG-rhG-CSF as primary versus secondary prophylaxis.

	Cost ($)	Risk of FN	QALY
**Base case**			
** Secondary prophylaxis**	16,313.43	17.7%	0.71
** Primary prophylaxis**	11,611.62	3.4%	0.73
** ICER**		Dominated	Dominated
**Risk for FN >20%**			
** Secondary prophylaxis**	17137.48	18.75%	0.70
** Primary prophylaxis**	11496.08	3.9%	0.74
** ICER**		Dominated	Dominated
**Risk for FN (10%-20%)**			
** Secondary prophylaxis**	15854.04	16.8%	0.71
** Primary prophylaxis**	11622.00	2.8%	0.74
** ICER**		Dominated	Dominated

FN, febrile neutropenia; QALY, quality-adjusted life-years; ICER, incremental cost-effectiveness ratio.

### Sensitivity Analysis

**FIGURE 2:**
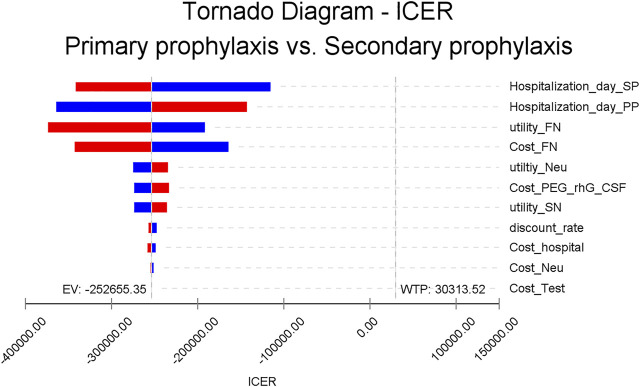
One-way sensitivity analysis. This diagram shows incremental cost-effectiveness ratio (ICER) of PP vs SP for different model input parameters. PEG-rhG-CSF, PEGylated recombinant human granulocyte–stimulating factor; FN, febrile neutropenia; SN, severe neutropenia; PP, primary prophylaxis; SP, secondary prophylaxis; Neu, neutropenia.

**FIGURE 3:**
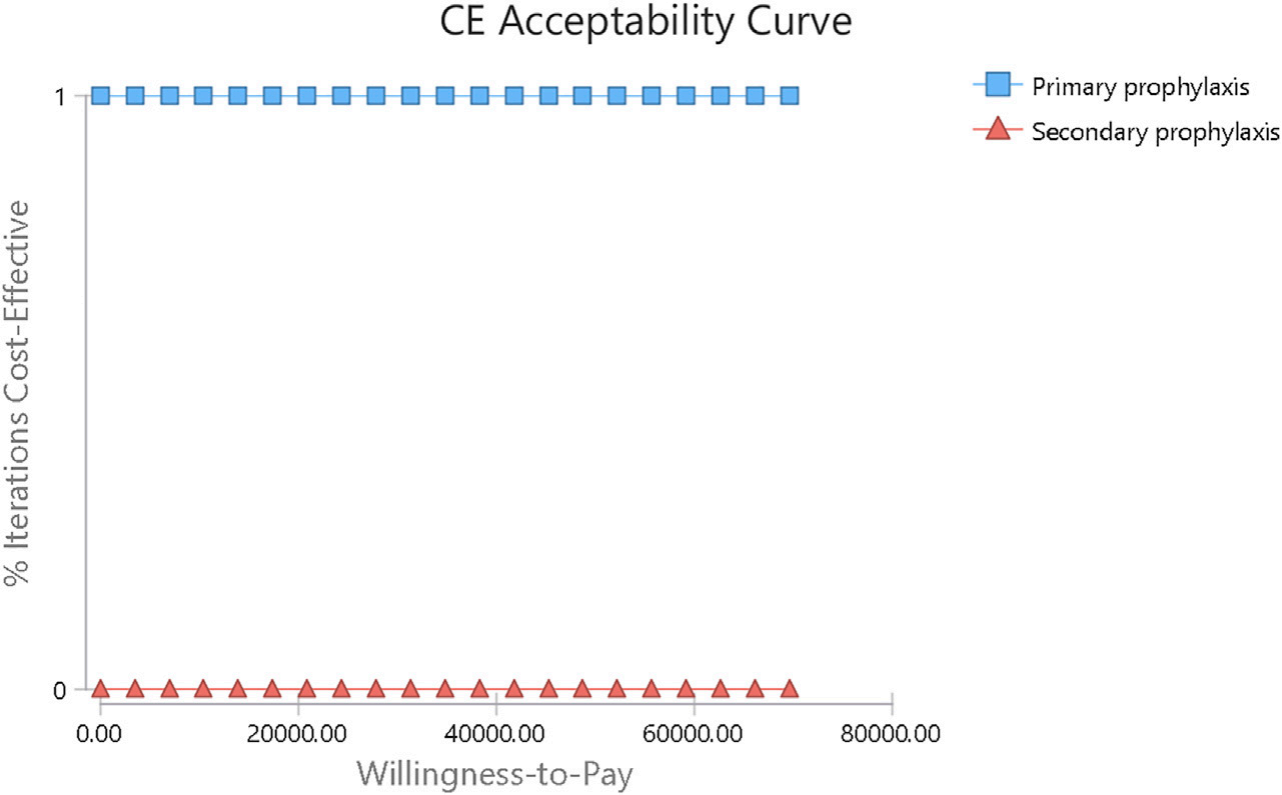
Acceptability curve for PEG-rhG-CSF primary versus secondary prophylaxis.

All one-way sensitivity investigation results are represented in Figure 2. One-way sensitivity analysis highlighted that this model’s reliability was more vulnerable to the mean hospitalization time frame in both study groups, together with the costs associated with FN clinical management, since both parameters have the greatest influence on ICER values. Notwithstanding, ICER was still identified to have a reduced value compared to WTP when all variable parameters shifted in value within the set range. Probabilistic sensitivity analysis consistently showed dominated results by PP, in comparison to SP (Figure 3).

## Discussion

In this study, Markov models were employed for assessing the cost-efficiency for PEG-rhG-CSF by retrospectively collecting and analyzing data on the effectiveness and cost of using this drug as PP and SP within three large hospitals. Compared with SP of PEG-rhG-CSF, this study revealed that PP of PEG-rhG-CSF not only increased the QLAY by 0.02 for patients with malignancies receiving intermediate- to high-risk FN chemotherapy, but there was also a reduction in FN events by 14.3%, together with a reduction in costs by $4,701.81. The analyzed model robustness was most vulnerable to the mean hospitalization time frame endured by both study groups, reflecting the impact of hospitalization costs on outcomes. One-way sensitivity was not used to analyze FN incidence since it was fixed in the model as a transition probability.

This study found that PP therapy incurred lower costs than SP therapy, having a lower FN incidence rate and a higher level of QALY. In the subgroup analysis, the median cost per QALY gained for PP patients was $15,535.24 and $15,705.41 in the FN high-and medium-risk groups, which was $8,946.87 and $6,624.22 lower than for secondary prevention patients, respectively. Such results were in conformity with reports from other nations focusing on this research niche (Hill et al., 2014). In Belgium, a study of PP and SP, including long-acting PEGylated rhG-CSF, found PP to be a cost-effective therapy for both breast cancer and non-Hodgkin’s lymphoma at a €30,000/QALY threshold (Fust et al., 2017). Similar conclusions regarding the cost-effectiveness of PP with long-acting PEGylated rhG-CSF versus SP have been reported in other studies in the United Kingdom, Singapore, and the United States (Gruschkus et al., 2011; Fust et al., 2014; Wang et al., 2016). Here, we expanded on past analyses by including different tumor types and obtained the incidence of FN for each cycle of the patients from multiple centers. We also analyzed the cost-effectiveness of PEG in FN intermediate- and high-risk chemotherapy regimens separately and therefore was pertinent for more complex clinical practice decision-making.

Overall, this investigation concludes that primary prophylaxis using G-CSF is a dominant strategy, when compared with secondary G-CSF-based prophylaxis for such oncology cases. Compared with PEG-rhG-CSF for secondary prophylaxis, albeit a more frequent use of PEG-rhG-CSF for primary prophylaxis results in a relatively higher cost, primary prophylaxis still proved to have enhanced cost-effectiveness potential against FN, also when FN-associated treatment costs are taken into account. However, unlike SP, PP does not fall within Chinese medical insurance provider coverage plans, leaving no reimbursement options for patients undergoing PP. Consequently, the findings from this study could provide empirical data to re-evaluate the reimbursement scheme.

As with all observational studies based on hospital information system, this study does carry limitations. In the first instance, these study data were based on past medical records, leading to several baseline variations (patient demographics, chemotherapy regimens, cancer type, the risk for febrile neutropenia, etc.) between the PP and SP groups. For the difference in the incidence of FN with chemotherapy regimens in the PP and SP groups, patients receiving moderate- and high-risk chemotherapy regimens in the two groups were analyzed separately and the findings were consistent with the overall population. In addition, regarding the age-adjusted Charlson comorbidity index and other factors that may be associated with the incidence of FN, as mentioned in the NCCN guidelines (16), we found no significant differences between the PP and SP groups. Overall, the cost of PEG⁃rhG⁃CSF is relatively expensive for patients in China, and only patients receiving secondary prevention are considered for reimbursement by medical insurance. We hope that more data will be available for further research following changes in health insurance policies, lower drug prices, or the inclusion of more institutions. Second, adverse events associated with PEG⁃rhG⁃CSF treatment were not considered in the model since PEG⁃rhG⁃CSF has relatively few adverse effects. Furthermore, the assessment of healthcare resource utilization, based on expert opinion, introduced a high degree of uncertainty into the model analysis. However, in the sensitivity analysis, all essential parameters were examined, and the model was stable when the parameters were varied. The probabilistic sensitivity analysis provided further evidence that PP is most likely a cost-effective option.

To our knowledge, this is the first real-world cost-effective study on PEG-rhG-CSF prophylaxis, from a Chinese payer’s perspective. In summary, for patients with tumors receiving chemotherapy regimens carrying >10% risk of incurring FN, primary prophylaxis with PEG-rhG-CSF reduces FN-related incidences, increases the likelihood of receiving a full dose, a full course of chemotherapy, and additional QALY benefits. Therefore, PEG-rhG-CSF is ideal for primary prophylaxis in terms of cost-effectiveness and has a reference value for adjusting health insurance reimbursement policies.

## Data Availability

The original contributions presented in the study are included in the article; further inquiries can be directed to the corresponding authors.
